# A pilot study of thiamin and folic acid in hemodialysis patients with cognitive impairment

**DOI:** 10.1080/0886022X.2021.1914656

**Published:** 2021-04-29

**Authors:** Renhua Lu, Yan Fang, Yijun Zhou, Miaolin Che, Jianxiao Shen, Qian Liu, Haifen Zhang, Shuting Pan, Yan Lin, Qin Wang, Shan Mou, Zhaohui Ni, Leyi Gu

**Affiliations:** a Department of Nephrology, Renji Hospital, School of Medicine, Shanghai Jiao Tong University, Shanghai, China; b Clinical Center for Investigation, Renji Hospital, School of Medicine, Shanghai Jiao Tong University, Shanghai, China; c Department of Neurology, Renji Hospital, School of Medicine, Shanghai Jiao Tong University, Shanghai, China

**Keywords:** End-stage renal disease, cognitive function, vitamins B, oxidative stress, survival

## Abstract

**Objective:**

This study aimed to explore the effectiveness of thiamin and folic acid supplementation on the improvement of the cognitive function in patients with maintenance hemodialysis.

**Method:**

In the present study, we randomly assigned patients undergoing hemodialysis who had the Montreal Cognitive Assessment (MoCA) score lower than 26 to treatment group (*n* = 25, thiamin 90 mg/day combined with folic acid 30 mg/day) or control group (*n* = 25, nonintervention). All subjects were followed up for 96 weeks. The primary outcome was the improvement of the MoCA score. The secondary outcomes included homocysteine level, survival and safety.

**Results:**

Patients in treatment group had an increase of the MoCA score from 21.95 ± 3.81 at baseline to 25.68 ± 1.96 at week 96 (*p* < 0.001, primary outcome), as compared with the MoCA score from 20.69 ± 3.40 to 19.62 ± 3.58 in control group. Thiamin combined with folic acid treatment also resulted in lower level of serum homocysteine in treatment group compare with control group at week 96 (*p* < 0.05, secondary outcome). 3 patients and 9 patients died during follow-up period in treatment and control group respectively (*p* = 0.048). The proportion of adverse events in treatment group was significantly lower than that in control group.

**Conclusion:**

Hemodialysis patients with cognitive impairment treated with thiamin and folic acid had a significant improvement in MoCA score.

## Introduction

End Stage Renal Disease (ESRD) is a public health issue of global concern. United States Renal Data System (USRDS) reported that the overall prevalence of ESRD in 2018 is 1,943 per million people in the United States [1]. The number of dialysis patients in Asia is expected to rise to 2.162 million by 2030 [2]. Cognitive Impairment (CI) is one of the common neurological complications in patients with ESRD [3]. Our previous single-center cohort observational study showed that the incidence of CI in patients with maintenance hemodialysis (MHD) was 51.6% and 3 times higher than that of non-Kidney disease patients [4]. MoCA score which has been confirmed in the previous literature [5,6] was used to determine cognitive impairment in MHD patients in this study. Furthermore, the 3-year survival rate of MHD patients with CI was significantly lower than that of patients with normal cognitive function [4].

An association between cognitive dysfunction and oxidative stress has been discussed [3]. Animal studies demonstrated that the cognitive function of ESRD mice which was assessed by the radial arm water maze test decreased compared with the sham-operated control mice. Furthermore, treatment with tempol (an antioxidant) in ESRD mice improved cognitive function compared to treatment with vehicle. Histological assessment suggested indicated increased oxidation in the hippocampal neurons and increased numbers of neurons undergoing apoptosis or necrosis, compared to control mice and those treated with tempol [7].

Recently, studies have found that thiamin, as a cofactor of transferenolase, plays an important role in reducing the production of reactive oxygen species (ROS) in the nervous system and reducing oxidative stress [8,9]. Folic acid has a direct antioxidant effect, which interacts with enzyme endothelial nitric oxide synthase (eNOS) and affects the bioavailability of nitric oxide cofactor [10]. Moreover, recent animal study have also found that oxidative stress index including malondialdehyde (MAD), protein carbonyl, 8-hydroxydeoxyguanosine (8-OHdG) and nitric oxide (NO) were closely related to thiamin and folic acid deficiency, leading to oxidative stress and cognitive impairment [11]. Clinical studies showed that both thiamin and folic acid supplementation can reduce the level of oxidative stress index including homocysteine in blood [10,12], thereby reducing oxidative stress and bringing benefits to the treatment of cardiovascular complications in MHD patients [12], particularly in those intensive treatment or with a drop in homocysteine levels of more than 20 percent [12]. However, recent systematic review and Meta-Analysis indicated that thiamin or folic acid alone cannot improves cognitive function healthy older people [13,14]. Therefore, we designed this pilot study to explore whether the intensive combination of these two vitamin B can improve cognitive function in MHD patients with CI. The dose of thiamin (90 mg/d) is refer to the treatment dose of Wernicke’s encephalopathy [15] and refeeding syndrome [16]. The dose of folic acid (30 mg/d) is according to the treatment of cardiovascular complications in MHD patients [12].

The present study conducted a randomized controlled pilot study to explore whether patients on MHD with CI can improve their MoCA score and survival by thiamin combined with folic acid.

## Materials and methods

### Study design and participant

**Figure 1.**
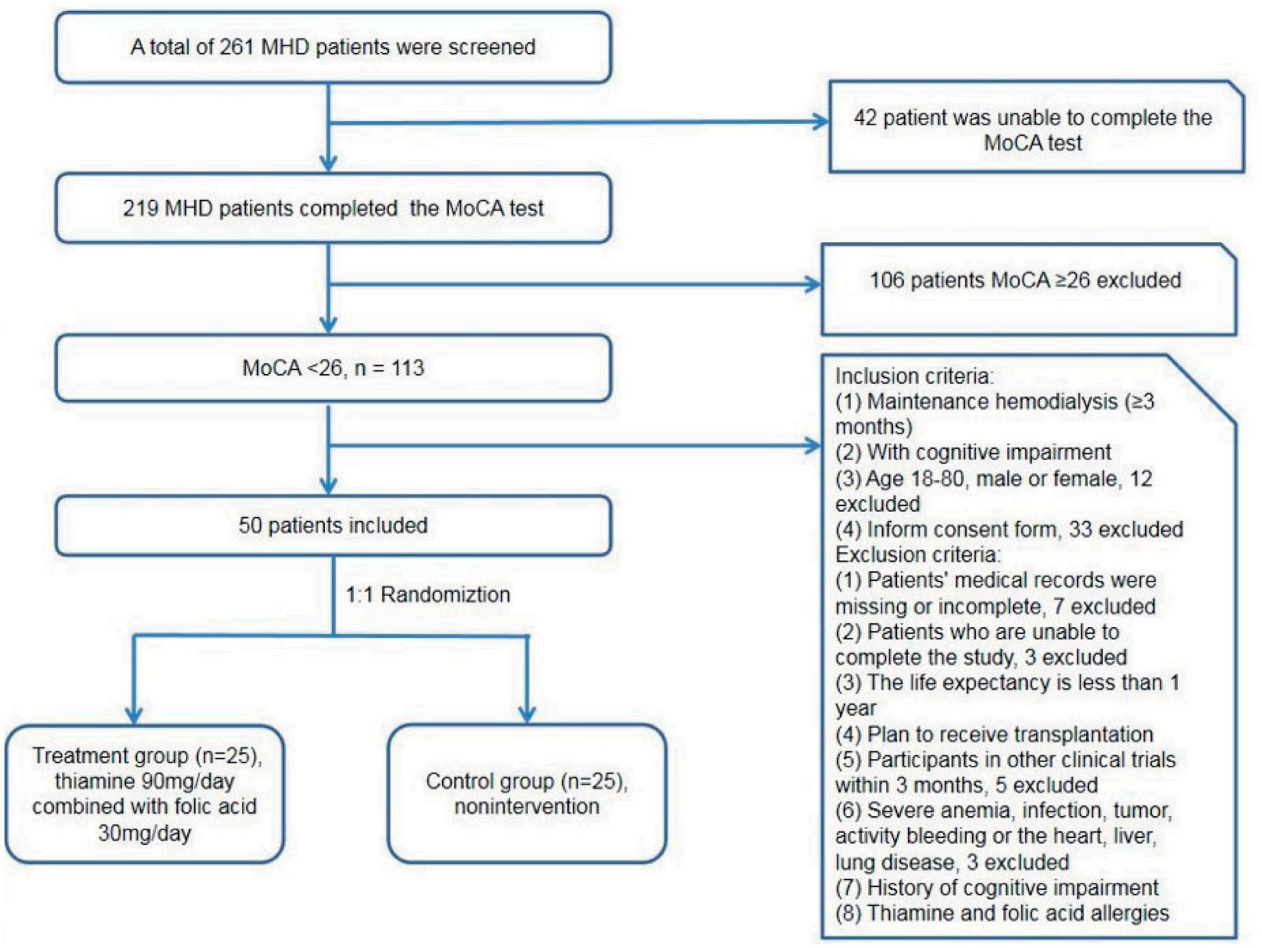
Patient inclusion and exclusion criteria.

This study was designed as a randomized, controlled, single-center study. Eligible patients were adults (18–80 years of age) with end-stage kidney disease who had been undergoing hemodialysis for at least three months and who had cognitive impairment (the MoCA score lower than 26 and <25 if the patients get education less than 12 years) [5]. The detail inclusion and exclusion criteria were showed in the Figure 1. Severe heart disease was defined as symptomatic heart failure, acute myocardial infarction, severe arrhythmias, sever valvular heart disease, etc. MoCA [6] was evaluated by professional neurologists to assess cognitive ability. The edition of the MoCA is beijing version 26 August 2006. Once the subjects are randomized, the neurologists did not know whether the subjects are in the treatment group or the control group. Every participant completed the MoCA test before or during the first hour of hemodialysis at baseline, week 48 and week 96. This trial was approved by Renji Hospital Ethical Committee, Shanghai Jiao Tong University School of Medicine. All procedures performed in studies involving human participants were in accordance with the ethical standards of the institutional and/or national research committee and and with the Helsinki Declaration of 1975 and its later amendments or comparable ethical standards. The study was registered on the Chinese Clinical Trial Registry in 2 August 2017 and URL of trial registry record is http://www.chictr.org.cn/index.aspx. Trial registration number is ChiCTR-IPR-17012210. Informed consent was obtained from all individual participants included in the study.

### Randomization

Randomization sequence was generated by an independent data manager using SAS (version 9.3) and stored within sealed opaque envelopes. The study physician opened an envelope to obtain the treatment assignment every time a patient was consented to enter the trial. Assignments were balanced in a 1:1 ratio between the two groups. No masking was applied.

### Data collection

Hemodialysis patients in Renji Hospital, School of Medicine, Shanghai Jiao Tong University were screened. Eligible patients assigned to treatment group received thiamin (Shanxi Fenhe Pharmaceutical Co. LTD, China) 90 mg/day combined with folic acid (Changzhou Pharmaceutical Co. LTD, China) 30 mg/day for 96 weeks, and those assigned to control group had nonintervention. All subjects were allowed stable doses of anticoagulant medicine, antihypertension medicine, hypoglycemic medicine and statins during the study period. The initiation of new improved cognitive medicine was prohibited. The subjects who signed the informed consent were grouped by the investigator according to the treatment allocation.

During the 96-weeks intervention period, urine volume and the following information of hemodialysis treatment were recorded: average weekly ultrafiltration (L/session), intradialytic hypotension (The definition of intradialytic hypotension is systolic pressure <90 mmHg or diastolic pressure <60 mmHg, accompanied by dizziness and other symptoms of hypotension during dialysis treatment within one week prior to follow-up), body weight of pre and post hemodialysis, blood pressure (BP) of pre and post hemodialysis.

### Efficacy outcomes

The primary outcome was the mean change from baseline at week 96 in the MoCA scores. The secondary outcomes included the mean change of serum homocysteine from baseline to week 96, survival and safety during follow-up.

### Estimated the serum thiamin, folic acid and homocysteine

Vitamin detector LINBIAO LK3000VI (Tianjin lanbiao electronic technology development co., LTD. China) and the original matching thiamin content determination kit were used to detect serum thiamin levels of two groups. Beckman Coulter’s UniCel DxI 800 Access Immunoassay System (Beckman Coulter, inc., United States) and the original folic acid assay kit were used to detect serum folic acid levels of two groups by immunoluminescence assay. The Hitachi 7600-020 automatic biochemical analyzer (Hitachi, Japan) and the corresponding homocysteine assay kit (Ningbo meicang biotechnology co., LTD. China) were used to detect serum homocysteine levels.

### Statistical analyses

The primary analysis only included patients with completed primary outcomes. The comparisons among MoCA scores and other secondary outcomes over time within subjects were conducted by using repeated measure ANOVA, and pairwise *t*-test with Bonferroni adjustment was performed for multiple comparisons. On the other hand, for comparisons between treatment and control group, simple one-way ANOVA or Wilcoxon rank sum test was used. The chisquare method was used for statistical analysis of the categorical data. For missing data in secondary efficacy outcomes, they were assumed to be missing at random and multiple imputation was conducted. Kaplan–Meier survival curve was used for survival analysis.

Variables of normal distribution were presented by means with SD while skewed ones were reported as the median and the inter-quartile ranges. Counting data were expressed as constituent ratios or percentages.

A *p* < 0.05 was considered statistically significant difference. Statistical analyses were conducted with SPSS (Version 20.0, SPSS Inc., Chicago, IL, USA).

## Results

### Baseline characteristics of patients

A total of 50 patients with MHD met the inclusion criteria and included in the present study. According to the study protocol, subjects were randomly divided 1:1 into treatment group (*n* = 25) and control group (*n* = 25) (Figure 1).

There was no significant difference in demographic data, medical history and hemodialysis data between treatment group and control group except ultrafiltration volume (*p* = 0.039; Table 1, Supplemental Table 1). Comparing the laboratory test, there was no significant difference except PH, bicarbonate, transferrin saturation (TAST), ferritin and Phosphorus (*p* < 0.05; Table 1, Supplemental Table 1). There was no significant difference in MoCA score between treatment group and control group (*p* = 0.076; Table 1).

**Table 1.** Comparison of baseline data between the treatment group and the control group.

	Treatment group (*n* = 25)	Control group (*n* = 25)	*p* Value
Age, years	66.16 ± 7.61	69.00 ± 10.80	0.287
Male, n (%)	18 (72)	19 (76)	0.747
History of smoking, n (%)	14 (56)	12 (48)	0.571
History of alcoholism, n (%)	11 (44)	7 (28)	0.239
History of drug abuse, n (%)	2 (8)	0 (0)	0.470
Education status (≥12 years, n [%])	11 (44)	14 (56)	0.369
Family history of CI, n(%)	1 (4)	0 (0)	1.000
Primary cause of MHD, n (%)			
Primary glomerulonephritis	5 (20)	7 (28)	0.508
Diabetic nephropathy	5 (20)	1 (4)	0.192
Hypertensive nephrosclerosis	3 (12)	6 (24)	0.462
ADPKD	1 (4)	1 (4)	1.000
Others	3 (12)	2 (8)	1.000
Unknown cause	8 (32)	8 (32)	1.000
Complication, n (%)			
Hypertension	24 (96)	23 (92)	1.000
Diabetes	9 (36)	5 (20)	0.208
Cardiovascular disease	8 (32)	5 (20)	0.333
Cerebrovascular disease	5 (20)	4 (16)	1.000
Cirrhosis	0 (0)	0 (0)	1.000
Chronic obstructive pulmonary disease	1 (4)	0 (0)	1.000
Urine, mL/day	0 (0,150)	0 (0,0)	0.230
Dialysis vintage, months	82.60 ± 51.90	107.40 ± 72.40	0.169
Dialysis duration (h/session)	4.00 ± 0.00	4.02 ± 0.10	0.322
Dialysis frequencies (time/week)	2.96 ± 0.20	2.92 ± 0.28	0.561
Intradialytic hypotension (patients, %)	13 (52)	12 (48)	0.777
Hb (g/L)	113.63 ± 15.54	112.59 ± 13.53	0.812
ALT (U/L)	11.13 ± 4.53	14.03 ± 7.97	0.131
AST (U/L)	12.42 ± 4.45	15.86 ± 6.89	0.054
TP (g/L)	72.14 ± 6.21	71.71 ± 5.02	0.797
Alb (g/L)	39.97 ± 2.02	39.47 ± 3.06	0.514
PH	7.28 ± 0.05	7.33 ± 0.04	0.000
HCO_3_^−^ (mmol/L)	18.39 ± 2.41	20.28 ± 2.23	0.009
K^+^ (mmol/L)	4.76 ± 0.86	4.50 ± 0.56	0.222
Na^+^ (mmol/L)	136.92 ± 3.30	136.82 ± 3.62	0.923
Ca (mmol/L)	2.25 ± 0.24	2.32 ± 0.21	0.293
P (mmol/L)	2.08 ± 0.63	1.57 ± 0.51	0.005
TC (mmol/L)	4.09 ± 0.96	3.83 ± 1.01	0.379
TG (mmol/L)	2.31 ± 1.39	2.07 ± 1.32	0.550
iPTH (pg/ml)	303.00 ± 212.08	215.04 ± 179.15	0.141
Glu (mmol/L)	6.15 ± 2.27	5.69 ± 52.27	0.496
CRP (mg/dl)	2.32 (1.12,5.13)	4.26 (1.77,7.77)	0.094
β_2_-MG (mg/L)	10.25 (7.58,12.05)	12.00 (8.20,43.40)	0.385
BNP (pg/ml)	219.50 (95.25,375.00)	198.50 (100.00,383.00)	0.809
spKt/V	1.58 ± 0.25	1.69 ± 0.11	0.063
Thiamin (nmol/L)	53.29 ± 7.12	52.79 ± 8.57	0.831
folic acid (ng/mL)	7.96 ± 2.10	8.77 ± 4.05	0.389
Homocysteine (μmol/L)	43.92 ± 21.05	46.82 ± 23.79	0.663
MoCA	22.08 ± 3.59	20.12 ± 4.02	0.076

Notes: The definition of drug abuse is drug dependence, no matter how much, such as sleeping pills.

### Primary analysis

MoCA scores at week 96 in treatment group and control group were 25.68 ± 1.96 and 19.63 ± 3.58 (*p* < 0.05; Table 2). In addition, MoCA scores increased significantly from baseline to 96 weeks in treatment group, but not control group (Table 2). The proportion of patients with MoCA scores greater than or equal to 26 was significantly higher in treatment group than that in control group at week 96 (72.7% *vs* 6.2%, *p* < 0.001; Supplemental Table 2).

**Table 2.** Comparison of MoCA scores, serum thiamin, folic acid and homocysteine in the control and treatment group at baseline, 48 weeks and 96 weeks of follow-up.

	Treatment group (*n* = 22)	Control group (*n* = 16)	*p* Value
MoCA			
Baseline	21.95 ± 3.81	20.69 ± 3.40	0.297
48 weeks	25.68 ± 2.40	20.00 ± 3.95	<0.001
96 weeks	25.68 ± 1.96	19.63 ± 3.58	<0.001
Comparison between 96 weeks and baseline, *p* value	<0.001	0.657	
Treatment difference over time, *p* value	<0.001	0.304	
Thiamin (nmol/L)			
Baseline	53.63 ± 7.33	51.98 ± 10.13	0.563
48 weeks	57.79 ± 6.09	54.90 ± 6.28	0.163
96 weeks	57.60 ± 5.86	52.87 ± 4.45	0.011
Comparison between 96 weeks and baseline, *p* value	0.168	1.000	
Treatment difference over time, *p* value	0.063	0.499	
Folic acid (ng/mL)			
Baseline	7.88 ± 2.18	8.39 ± 2.63	0.519
48 weeks	21.94 ± 4.85	8.09 ± 4.62	<0.001
96 weeks	18.35 ± 7.94	7.3 ± 4.83	<0.001
Comparison between 96 weeks and baseline, *p* value	<0.001	0.870	
Treatment difference over time, *p* value	<0.001	0.719	
Homocysteine (μmol/L)			
Baseline	43.55 ± 21.81	48.72 ± 27.43	0.522
48 weeks	33.64 ± 13.04	41.36 ± 13.34	0.083
96 weeks	32.58 ± 13.42	45.69 ± 18.54	0.016
Comparison between 96 weeks and baseline, *p* value	0.111	1.000	
Treatment difference over time, *p* value	0.063	0.594	

Notes: The normal range for blood thiamin concentration is approximately 70–180 nmol/L [18] and the normal range for serum levels of folate acid is approximately 2.7–17 ng/ml [19] in the general population.

### Secondary analysis

Seven items of MoCA score were compared between the treatment group at baseline and the 96 weeks of follow-up. We found that the item of delayed recall was improved by thiamin and folic acid treatment significantly (Supplemental Table 3).

At 96 weeks of follow-up, the level of serum homocysteine in treatment group and control group was 32.58 ± 13.42 μmol/L and 45.69 ± 18.54 μmol/L, *p* = 0.016. We also found that the levels of serum thiamin and folic acid in treatment group were significantly higher than those in control group (Table 2).

In addition, the level of folic acid, but not serum thiamin or homocysteine had significantly changed over time in treatment group (Table 2).

There was no significant difference in hemodialysis data and laboratory test between treatment group and control group at 48 weeks or 96 weeks of follow-up (Supplemental Tables 4 and 5).

**Figure 2.**
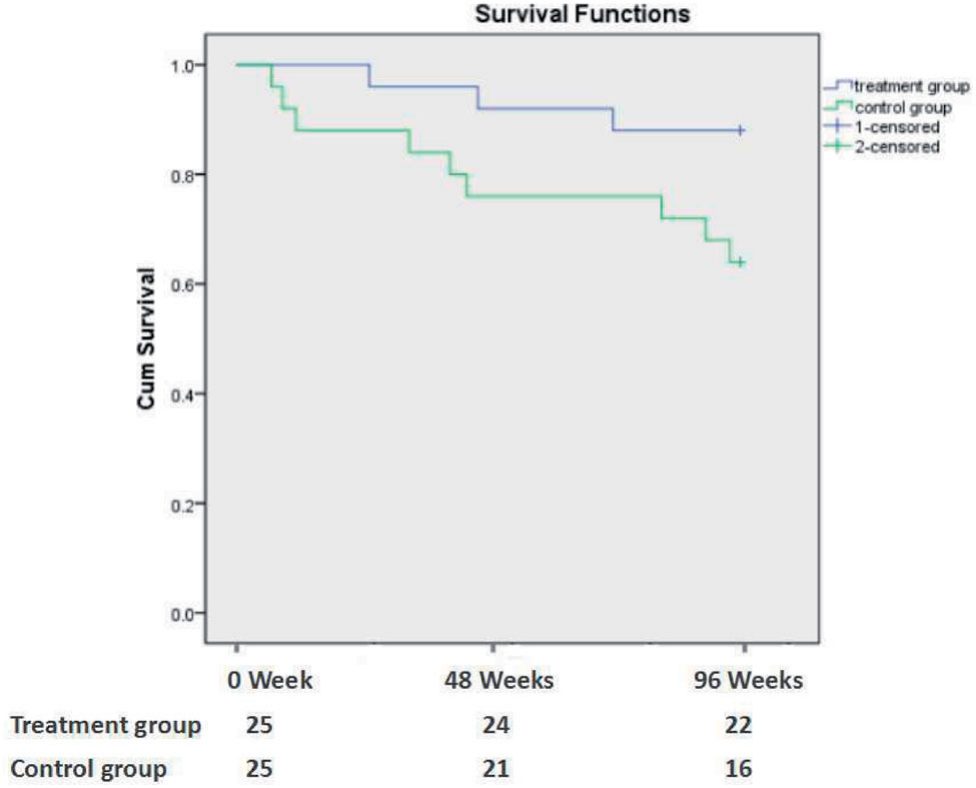
The Kaplan–Meier patient survival analysis between the treatment group and the control group (*p* = 0.048).

A total of 12 patients died during a follow-up of 96 weeks. The mortality rate in treatment group and control group was 12.0% and 36.0%, *p* = 0.047. The Kaplan–Meier survival curve analysis showed that the survival rate of treatment group was lower than that of control group (*p* = 0.048; Figure 2). The causes of death were cardiovascular and cerebrovascular diseases (9 cases, 75.0%), malignant tumors (2 cases, 16.7%), and infection (1 case, 8.3%) (Table 3).

**Table 3.** Comparison of major causes of death between treatment group and control group.

	Treatment group (*n* = 3)	Control group (*n* = 9)
Cardiovascular and cerebrovascular events, n (%)	1 (33.3%)	8 (88.9%)
Malignant tumor, n (%)	2 (66.7%)	0 (0%)
Infection, n (%)	0 (0%)	1 (11.1%)

There were 33 (66%) adverse events (AE) occurred during follow-up period. In treatment group, there were 7 events (21.2%), including 3 malignant tumor, 2 cardiovascular and cerebrovascular events, 1 heart failure and 1 hypotension. In control group, there were 26 AE (78.8%), including 12 cardiovascular and cerebrovascular events, 3 heart failure, 3 infection, 3 internal fistula dysfunction, 2 upper gastrointestinal bleeding and 1 hypotension. The proportion of AE in control group was significantly higher than that in treatment group (*p* = 0.000). Among them, cardiovascular and cerebrovascular events in treatment group were significantly lower than those in control group (*p* = 0.005).

## Discussion

In the present pilot study, we found that the MoCA score of treatment group was significantly improved, implicating that supplementation with thiamin and folic acid may improve the cognitive function of patients undergoing MHD with CI. The survival of patients in treatment group was significantly improved compared with that in control group. The proportion of adverse events in the control group was significantly higher than that in the treatment group, especially in cardiovascular and cerebrovascular events.

The present study pointed out that after 96 weeks of thiamin (90 mg/day) and folic acid (30 mg/day) supplementation, the MoCA score of the treatment group was significantly higher than that of control group, implicating the improvement of cognitive function. Recently, an animal study in mice demonstrated that thiamin deficiency decreased activity of antioxidants and increased activity of malondialdehyde (MAD), protein carbonyl, 8-hydroxydeoxyguanosine (8-OHdG) and nitric oxide (NO) in the cerebral cortex and hippocampus, leading to oxidative stress and cognitive impairment [11]. Meanwhile, an experimental mouse model simulating the effects of exposure to methotrexate on behavior and cognitive function found acute decrease in serum and CSF levels of folate acid, leading to oxidative stress and displayed cognitive [17]. This may be due to thiamin, as a cofactor of ketolase, can act as an oxygen free radical scavenger and play an important role in reducing the production of reactive oxygen species (ROS) in the nervous system and alleviating oxidative stress [11]. Another reason is the direct antioxidant effect of folic acid, which interacts with endothelial nitric oxide synthase (eNOS) to affect the bioavailability of NO cofactors. Moreover, folic acid is essential for the metabolism of homocysteine into methionine, which can reduce the homocysteine level of MHD patients, thus relieving oxidative stress [10]. However, there is insufficient evidence to support supplementation with thiamin or folic acid alone can improve cognitive functioning in healthy older people or non-chronic kidney diseases (CKD) older adults [13,14]. Therefore, we designed this pilot study to explore whether the combination of these two vitamin B can improve cognitive function in MHD patients with CI.

Furthermore, the normal range for blood thiamin concentration is approximately 70–180 nmol/L in the general population [18]. However, in MHD patients, the level of blood thiamin concentration is lower than general population which has been founded in our study. Because thiamin is a water-soluble vitamin, the absorption is affected by uremia toxins. Moreover, it will be cleared by dialysis, especially in patients receiving high-flux dialysis. After 96 weeks of thiamin supplementation, blood thiamin concentration rose, but did not reach the normal range, which may be related to the decreased expression of thiamin transporter 1 and thiamin transporter 2 caused by uremia environment [19] or a higher dose of thiamin may be needed. Further research is needed to confirm this. Moreover, the normal range for serum levels of folate is approximately 2.7–17 ng/ml in the general population [19]. However, in MHD patients, the serum levels of folate are at the low end of the normal range which has been founded in our study. After 96 weeks of folate supplementation, serum levels of folate were above the normal range,and this is exactly what our research needs to achieve.

Homocysteine is not only a uremia toxin but also a biomarker of oxidative stress in patients with MHD. Many observational studies have suggested that elevated blood homocysteine levels are strongly associated with CI [20–22]. Moreover, previous studies have found that there are many mechanisms that cause elevated homocysteine levels in MHD patients including deficiency of vitamins B, especially thiamin and folate acid [23]. In the present study, blood homocysteine levels were significantly higher than the normal range in both groups at baseline, suggesting the presence of oxidative stress in patients of MHD with CI [24]. Compared with baseline, homocysteine did not decrease significantly (*p* = 0.063) at week 96. Although, serum homocysteine level at week 96 in treatment group was lower than that in control group, we cannot conclude thiamin combined with folic acid treatment improve MoCA scores dependent on decreasing homocysteine. A new study with larger sample size and longer follow-up time should be performed to clarify the role of serum homocysteine.

It is worth mentioning that the survival rate of MHD patients with CI was significantly lower than that of patients with normal cognitive function, suggesting that CI is a risk factor for death of MHD patients [4]. Recent COGNITIVE-HD studies have also confirmed this phenomenon [25], therefore, it is urgent to find effective methods or measures to treat or improve the concurrent CI of MHD. In this study, Kaplan–Meier survival curve analysis indicated that the survival rate in treatment group was significantly higher than that in control group. The main cause of death in the two groups is cardiovascular and cerebrovascular events (9 cases, accounting for 75%), including 8 cases (88.9%) in the control group and 1 case (33.3%) in the treatment group. We think that abundant thiamin combined with folic acid treatment does not only improve MoCA scores but also decrease the risk of cardiovascular and cerebrovascular disease in patients undergoing hemodialysis. The possible reason is that oxidative stress is one of the common pathogenesis of cognitive impairment and cardiovascular and cerebrovascular diseases in MHD patients [3]. Therefore, the application of vitamins B which could inhibit oxidative stress, especially in the brain, may improve cognitive function and prevent the progression of cardiovascular and cerebrovascular diseases at the same time.

Several limitations of our study should be considered. First, this study was not designed to use a blind method and placebo control. It may lead to the generation of psychological bias and influence the MoCA scores. Furthermore, the sample size is small, which will lead to insufficient grasp in statistics and may cause false negative results. Moreover, we only used MoCA score as the basis for judging cognitive functions, which was highly subjective and could not cover all the assessments of cognitive functions. Future studies need to include more scoring criteria such as neuropsychological battery of 10 tests [26], and even imaging tests such as functional magnetic resonance imaging [27], to accurately judge cognitive function. Finally, patients in treatment group were given two medicines, it was difficult to identify which one played a key role.

In conclusion, supplementation of thiamin (90 mg/day) combined with folic acid (30 mg/day) can improve MoCA scores and survival of MHD patients with CI.

## Supplementary Material

Supplemental Material

Supplemental Material

Supplemental Material

Supplemental Material

Supplemental Material

## Data Availability

The data analyzed in the current study are available from the corresponding author on reasonable request. Raw data retained at the Clinical Center for Investigation, Renji Hospital (RJDBLK2020-03-N13001).
