# Supplementary material for: A pilot study of thiamin and folic acid in hemodialysis patients with cognitive impairment
Source: Ren Fail. 2021 Apr 29;43(1):766–73. doi: 10.1080/0886022X.2021.1914656 (PMC8901284; doi:10.1080/0886022X.2021.1914656)
Supplement: Supplemental Material [file IRNF_A_1914656_SM8632.pdf]

Supplemental Table 1. Comparison of baseline data between the treatment group and the control group

	Treatment group (n=25)	Control group (n=25)	p value
Height, cm	166.36±7.31	166.52±6.61	0.936
Income (≥30,000 Yuan, n [%])	18（72）	22（88）	0.157
Medical costs (10,000 Yuan/year)	0.93±0.83	0.70±0.44	0.265
Working status, n (%)	25（100）	24（96）	1.000
Active vitamin D therapy, n (%)	12（48）	10（40）	0.569
Intravenous iron therapy, n (%)	8（32）	8（32）	1.000
Dialysis modality (HD, %)	19（76）	19（76）	1.000
Vascular access (fistula, %)	25（100）	25（100）	1.000
Average ultrafiltration (L/session)	2.68±0.91	2.25±0.46	0.039
Low molecular heparin (%)	11（44）	11（44）	1.000
Pre-dialysis weight, kg	66.31±10.06	61.98±8.70	0.110
Post-dialysis weight, kg	64.19±9.65	59.77±8.84	0.097
Pre-dialysis systolic pressure, mmHg	143.64±30.88	140.48±23.37	0.685
Post-dialysis systolic pressure, mmHg	139.84±24.80	135.16±23.67	0.498
Pre-dialysis diastolic pressure, mmHg	71.16±10.79	69.36±13.20	0.600
Post-dialysis diastolic pressure, mmHg	74.80±12.49	68.88±12.75	0.104
Pre-dialysis heart rate, BPM	77.44±8.58	77.36±10.83	0.977
Post-dialysis heart rate, BPM	78.80±10.46	74.36±12.37	0.177
WBC ($*10^9$)	7.14±2.40	7.12±1.49	0.983
Plt ($*10^9$)	185.00±71.50	206.00±62.29	0.296
TB (mmol/L)	5.60±1.77	6.06±2.63	0.489
Cl^- (mmol/L)	103.71±5.65	102.82±4.52	0.561
LDL (mmol/L)	2.54±0.94	2.28±0.90	0.339
HDL (mmol/L)	0.91±0.25	0.95±0.20	0.535
Ferritin (ng/ml)	108.20（40.68,323.42）	227.50（122.35,649.88）	0.016
TSAT (%)	24.81±10.22	37.74±18.72	0.007
TSH (mIU/L)	1.58±1.31	1.48±0.74	0.753
