# Supplementary material for: A pilot study of thiamin and folic acid in hemodialysis patients with cognitive impairment
Source: Ren Fail. 2021 Apr 29;43(1):766–73. doi: 10.1080/0886022X.2021.1914656 (PMC8901284; doi:10.1080/0886022X.2021.1914656)
Supplement: Supplemental Material [file IRNF_A_1914656_SM8631.pdf]

Supplemental Table 2. Comparison of the proportion of patients with MoCA scores≥26 points at 48 and 96 weeks of follow-up between the treatment group and the control group

	Treatment group		Control group	
	48weeks (n=22)	96weeks (n=22)	48weeks (n=16)	96weeks (n=16)
MoCA≥26（%）	11（50.0）*	16（72.7）#	1（6.2）	1（6.2）
MoCA<26（%）	11（50.0）	6（27.3）	15（93.8）	15（93.8）

*48 weeks of follow-up in treatment group *vs* 48 weeks of follow-up in control group, p=0.012

#96 weeks of follow-up in treatment group *vs* 96 weeks of follow-up in control group, p<0.001
