# Supplementary material for: A pilot study of thiamin and folic acid in hemodialysis patients with cognitive impairment
Source: Ren Fail. 2021 Apr 29;43(1):766–73. doi: 10.1080/0886022X.2021.1914656 (PMC8901284; doi:10.1080/0886022X.2021.1914656)
Supplement: Supplemental Material [file IRNF_A_1914656_SM8623.pdf]

Supplemental Table 3. Comparison of seven items of MoCA score at baseline and the 96 weeks of follow-up in the treatment group.

Items of MoCA (sore)	T0(n=22)	T4(n=22)	p-value	Alpha after Bonferroni adjustment
Visuospatial/executive(5)	4.09±1.02	4.27±0.88	0.296	0.007
Naming(3)	2.82±0.40	2.73±0.46	0.162	0.007
Attention(6)	4.27±1.08	4.64±0.85	0.073	0.007
Language(3)	1.86±0.56	2.09±0.68	0.135	0.007
Abstraction(2)	1.23±0.81	1.50±0.67	0.030	0.007
Delayed recall(5)	1.82±1.18	4.41±0.80	0.000*	0.007
Orientation(6)	5.95±0.21	5.95±0.21	1.000	0.007

*Bonferroni adjustment of the items of the MoCA shows delayed recall was improved by thiamine and folic acid treatment significantly
