# Supplementary material for: A pilot study of thiamin and folic acid in hemodialysis patients with cognitive impairment
Source: Ren Fail. 2021 Apr 29;43(1):766–73. doi: 10.1080/0886022X.2021.1914656 (PMC8901284; doi:10.1080/0886022X.2021.1914656)
Supplement: Supplemental Material [file IRNF_A_1914656_SM8621.pdf]

Supplemental Table 4. Comparison of haemodialysis data between the treatment group and the control group at 48weeks 96 weeks of follow-up

	Treatment group		Control group	
	48 weeks （n=24）	96 weeks （n=22）	48 weeks （n=21）	96 weeks （n=16）
Dialysis duration (h/ session)	4.04±0.20	4.00±0.00	4.00±0.00	4.00±0.00
Dialysis frequencies (time/week)	2.96±0.20	3.0±0.00	2.95±0.22	2.94±0.25
Dialysis modality (HD, %)	12（50）	11（50）	11（52.4）	8（50）
Vascular access (fistula, %)	24（100）	22（100）	21（100）	16（100）
Average ultrafiltration (L/session)	2.50±0.77	2.66±0.66	2.23±0.57	2.41±0.88
Intradialytic hypotension (patients, %)	12（50）	10（45.5）	10（47.6）	7（43.8）
Low molecular heparin (%)	9（37.5）	22（100）	7（33.3）	16（100）
Pre-dialysis weight, kg	64.44±9.96	62.22±10.41	61.04±7.98	61.54±9.81
Post-dialysis weight, kg	62.41±9.56	59.94±10.21	58.99±7.74	59.36±9.43
Pre-dialysis systolic pressure, mmHg	148.13±25.96	137.5±26.9	141.2±23.02	146.94±26.74
Post-dialysis systolic pressure, mmHg	135.63±21.59	132.23±24.18	144.50±33.95	129.19±22.81

Pre-dialysis diastolic pressure, mmHg	69.33±13.42	67.82±9.56	71.80±12.58	70.75±13.13
Post-dialysis diastolic pressure, mmHg	69.25±12.26	67.55±10.59	71.90±17.73	64.75±14.93
Pre-dialysis heart rate, BPM	77.96±9.20	76.05±9.65	74.15±10.57	74.63±12.14
Post-dialysis heart rate, BPM	78.33±12.56	73.64±11.76	75.15±11.08	75.31±9.0
