# Supplementary material for: A pilot study of thiamin and folic acid in hemodialysis patients with cognitive impairment
Source: Ren Fail. 2021 Apr 29;43(1):766–73. doi: 10.1080/0886022X.2021.1914656 (PMC8901284; doi:10.1080/0886022X.2021.1914656)
Supplement: Supplemental Material [file IRNF_A_1914656_SM8604.pdf]

**Supplemental Table 5. Comparison of laboratory examination between the treatment group and the control group at 48weeks 96 weeks of follow-up**

| | Treatment group | | Control group | |
|---|---|---|---|---|
| | 48 weeks（n=24） | 96 weeks（n=22） | 48weeks（n=21） | 96weeks（n=16） |
| WBC ($*10^9$) | 6.33±2.12 | 6.40±1.52 | 6.56±1.37 | 5.67±1.01 |
| Hb (g/L) | 120.04±23.34 | 115.05±15.42 | 106.38±16.42 | 110.38±15.61 |
| Plt ($*10^9$) | 165.65±65.89 | 179.82±76.47 | 210.75±53.38 | 188.88±53.19 |
| ALT (U/L) | 11.10±5.93 | 10.23±6.32 | 14.25±20.82 | 13.94±12.36 |
| AST (U/L) | 12.68±4.06 | 11.9±6.8 | 14.38±11.66 | 13.63±8.65 |
| TP (g/L) | 69.09±5.23 | 67.66±5.36 | 71.32±4.62 | 66.85±7.52 |
| TB (mmol/L) | 5.74±1.63 | 5.19±2.37 | 7.27±4.50 | 6.37±2.92 |
| Alb (g/L) | 41.16±3.48 | 38.03±3.67 | 40.60±3.78 | 36.76±5.42 |
| PH | 7.43±0.03 | 7.39±0.05 | 7.44±0.03 | 7.39±0.05 |
| $HCO_3^-$ (mmol/L) | 26.13±1.01 | 24.3±2.99 | 25.80±1.41 | 24.15±3.04 |
| $K^+$ (mmol/L) | 3.13±0.53 | 3.65±0.99 | 3.34±0.61 | 3.49±0.68 |
| $Na^+$ (mmol/L) | 136.05±3.20 | 135.77±2.16 | 136.06±4.60 | 138.0±2.73 |
| $Cl^-$ (mmol/L) | 103.00±5.12 | 100.66±3.24 | 100.19±6.52 | 102.5±10.04 |
| Ca (mmol/L) | 2.36±0.25 | 2.37±0.28 | 2.37±0.29 | 2.34±0.30 |
| P (mmol/L) | 1.64±0.46 | 1.62±0.53 | 1.69±0.64 | 1.77±0.59 |
| TC (mmol/L) | 4.33±1.08 | 4.26±1.46 | 4.03±1.21 | 4.05±1.20 |
| TG (mmol/L) | 2.64±1.90 | 2.43±1.45 | 1.97±1.44 | 2.04±1.11 |
| LDL (mmol/L) | 2.58±0.94 | 2.56±1.21 | 2.30±0.88 | 2.37±1.01 |
| HDL (mmol/L) | 1.00±0.24 | 0.94±0.25 | 1.06±0.31 | 0.95±0.30 |
| iPTH (pg/ml) | 331.25±246.60 | 345.1±250.93 | 289.06±234.28 | 274.38±235.19 |
| Ferritin (ng/ml) | 190.4(84.75,635.45) | 310.75(103.0,737.15) | 212.3(31.35, 543.73) | 257.6(51.45,483.48) |

| | | | | |
|---|---|---|---|---|
| TSAT (%) | 33.39±13.90 | 30.43±12.60 | 31.54±15.37 | 29.67±14.23 |
| Glu (mmol/L) | 7.25±2.42 | 6.07±3.04 | 7.08±3.08 | 6.14±3.07 |
| CRP (mg/dl) | 3.12(1.15,10.33) | 3.16(1.05,5.72) | 7.12(1.82,17.0) | 3.74(1.64,9.3) |
| $\beta_2$-MG (mg/L) | 7.7(6.25,12.25) | 9.75(8.3,29.68) | 9.0(7.85,41.48) | 14.0(9.23,42.68) |
| BNP(pg/ml) | 179.0(115.0,529.0) | 237.0(120.0,431.0) | 248.5(96.0,507.0) | 383.0(94.25,910.0) |
| spKt/V | 1.71±0.25 | 1.73±0.25 | 1.66±0.22 | 1.60±0.19 |
